# Applying Personal Genetic Data to Injury Risk Assessment in Athletes

**DOI:** 10.1371/journal.pone.0122676

**Published:** 2015-04-28

**Authors:** Gabrielle T. Goodlin, Andrew K. Roos, Thomas R. Roos, Claire Hawkins, Sydney Beache, Stephen Baur, Stuart K. Kim

**Affiliations:** 1 Departments of Developmental Biology and Genetics, Stanford University Medical Center, Stanford, CA, 94305, United States of America; 2 Division of Epidemiology, Department of Health Research and Policy, Stanford University School of Medicine, Stanford, CA, 94305, United States of America; 3 Department of Human Biology, Stanford University, Stanford, CA, 94305, United States of America; Universidad Europea de Madrid, SPAIN

## Abstract

Recent studies have identified genetic markers associated with risk for certain sports-related injuries and performance-related conditions, with the hope that these markers could be used by individual athletes to personalize their training and diet regimens. We found that we could greatly expand the knowledge base of sports genetic information by using published data originally found in health and disease studies. For example, the results from large genome-wide association studies for low bone mineral density in elderly women can be re-purposed for low bone mineral density in young endurance athletes. In total, we found 124 single-nucleotide polymorphisms associated with: anterior cruciate ligament tear, Achilles tendon injury, low bone mineral density and stress fracture, osteoarthritis, vitamin/mineral deficiencies, and sickle cell trait. Of these single nucleotide polymorphisms, 91% have not previously been used in sports genetics.

We conducted a pilot program on fourteen triathletes using this expanded knowledge base of genetic variants associated with sports injury. These athletes were genotyped and educated about how their individual genetic make-up affected their personal risk profile during an hour-long personal consultation. Overall, participants were favorable of the program, found it informative, and most acted upon their genetic results.

This pilot program shows that recent genetic research provides valuable information to help reduce sports injuries and to optimize nutrition. There are many genetic studies for health and disease that can be mined to provide useful information to athletes about their individual risk for relevant injuries.

## Introduction

The regular demands of training and competition during a season make professional, collegiate, and recreational athletes alike highly susceptible to injury. From 1988 through 2003, the incidence rate of injuries in NCAA athletes was 15.47 per 1,000 athlete exposures[1]. Recreational distance running causes high numbers of injuries, with incidence rates estimated between 30% and 75% per year[2,3]. Similarly, up to 74.8% of age-group triathletes that participate in Ironman-distance races will be injured at least once each training season[4,5]. Avoiding injuries and staying healthy is key to a team or individual athlete’s success[6].

Recent genetic research has revealed specific markers associated with increased risk for sports injuries and performance-related conditions[7,8,9]. Use of this genetic information may aid in the development of tailored injury prevention program for athletes, which could provide a new edge for successful competition[10]. For example, 100 genetic loci involved in sports performance were analyzed for a professional soccer team in the English Premier League, and the English Institute of Sport expressed interest in providing genetic testing to Britain’s Olympic athletes[11]. As of 2012, at least thirteen direct-to-consumer genomic companies offer genetic testing to athletes and pilot studies of the genomic profiling of soccer players is underway[10,12,13].

In this paper, we examine the possibility of applying genetic knowledge about health and disease to predicting risk of sports injury in athletes. We performed a literature search on genetic studies in three categories of health that had not been previously used in the context of sports genetics: stress fracture, vitamin and mineral deficiencies, and osteoarthritis. We found a large number of DNA polymorphisms (113) in these categories, along with 11 polymorphisms that had been previously associated with athletic injuries (anterior cruciate ligament rupture, Achilles tendon injury, and sickle cell trait). We chose to focus on injuries whose risk factors could be addressed by modifications in training or diet, rather than non-actionable injuries, such as concussions. Through our literature search, we identified 113 SNPs that have previously not been discussed in relation to sports injuries and performance. A new sports genetic database was constructed, and used to help customize injury prevention and training for fourteen athletes. Fourteen elite triathletes from the Stanford University Triathlon Team were genotyped and given information that could lead to individualized changes in training routines and injury prevention programs.

## Materials and Methods

Genome-wide association studies (GWAS) from the NHGRI GWAS Catalog and candidate gene studies from PubMed (MEDLINE) were selected for their relevance to athletic health: bone mineral density, osteoarthritis, calcium levels, vitamin E, vitamin D, vitamin B12, folate pathway vitamin levels, homocysteine, and phytosterol levels. Single nucleotide polymorphisms (SNPs) from genome-wide studies that reached genome-wide significance were accepted, along with SNPs from candidate gene studies. The studies were rated according to the criteria established for the assessment of cumulative evidence on genetic associations and effect size estimates were based on odds ratios reported for published SNPs associated with each category (OR 1.0–1.3 = small effects, 1.3–2.0 = medium effects, and >2.0 = large effects)[14].

We wanted to test our sports genetics database on a small set of elite athletes and recruited nineteen athletes on the Stanford University Triathlon Team based on their commitment to training and athletic ability (Table 1). Athletic ability was defined as those who qualified to compete at the 2012 USAT Collegiate National Championship or equivalent. Athletic injuries were defined according to the NCAA standard of missing at least one day of practice or competition due to a diagnosed injury[1]. At Stanford, triathlon is a club sport that is scored by individual performances, which minimizes ethical complications arising from genetic discrimination affecting athletic scholarships or starting lineups. This set of elite athletes is too small to perform a rigorous trial to show the effectiveness of the sports genetics program in reducing injury incidence. Our goal was to simply observe their responses to the new genetic knowledge. Of the nineteen recruited, fourteen consented to participate in this pilot program and athletes were genotyped through 23andMe, a direct-to-consumer company. The raw data file was used to identify the genotype at rsIDs that were found to be associated with sports injury risk based on our data mining of published literature. We conducted an hour-long personal consultation for each athlete, during which we explained how their personal genetic data affected their risk in each of the six sports injury categories. If an athlete was at risk in any category, we provided information about interventions to reduce that risk.

**Table 1:** Summary of Athlete Cohort.

	N (%)	Age (yrs)	Varsity (%) *	Training (hrs/week) **	Injury 2012 (%) ***	Injury 2013 (%) ***
**All**	14 (100%)	24.8 [20–38]	85.7%	13.1 [5–20]	71.4%	33.3%
**Male**	9 (64.3%)	25.6 [20–38]	88.9%	-	66.6%	22.2%
**Female**	5 (35.7%)	23.6 [20–29]	80.0%	-	80.0%	40.0%

Note: Numbers listed are the mean from all athletes in the cohort and numbers in brackets are the min-max range for those characteristics.

*: Varsity status is defined as whether or not the athlete participated on an NCAA Div 1, II, or III Varsity level team (swimming, cross-country, track-and-field, crew) for at least 1 year prior to being on the Stanford Triathlon Team.

**: Self-reported average training hours per week during the collegiate season from Sept 1^st^, 2012 to May 1^st^, 2013. Training was periodized, includes off days plus rest weeks, includes structured team plus individual workouts, and is not limited by discipline.

***: Injury status is defined as any athlete who was limited in their training, could not participate in team workouts, or was unable to race for at least one day due to a diagnosed injury from Sept 1^st^ to Aug 31^st^ of each season.

Three and twelve months after their consultation meeting, athletes were sent surveys using REDCap (Research Electronic Data Capture) software[15] to assess genetic information sharing, psychological effects, behavioral changes in training and/or diet, and to track injuries. Ten and thirteen out of fourteen participants filled out the three-month and twelve-month surveys, respectively (S1 File).

### Ethics Statement

The pilot program protocol was approved by the Research Compliance Office at Stanford University (IRB Protocol 24576). Written informed consent was obtained from all athletes to participate in the study, as well as verbal consent to use their deidentified information for study purposes.

## Results

**Fig 1:**
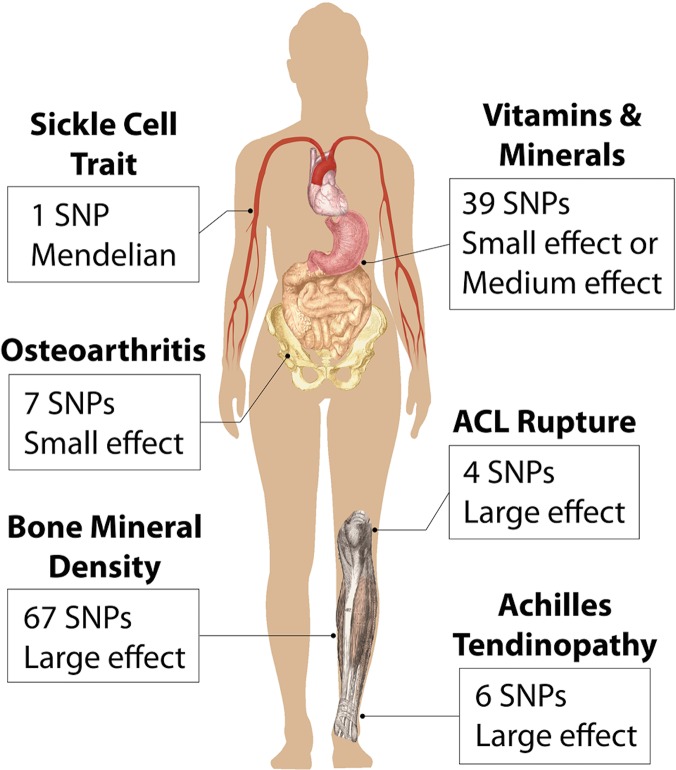
Areas of Interest for Genetic Markers. Six sports related categories were tested in athletes that relate to different injuries or attributes in different locations of the human body. For each category we list the number of associated single nucleotide polymorphisms (SNPs) that we reported on as well as the overall effect size, based on odds ratios or β-coefficients, for having a genetic risk in that category.

The literature review identified a total of 124 SNPs in the following categories: bone mineral density (BMD), osteoarthritis, vitamin and mineral deficiencies, ACL rupture, Achilles tendon injury, and sickle cell trait (SCT) (Fig 1).

**Fig 2:**
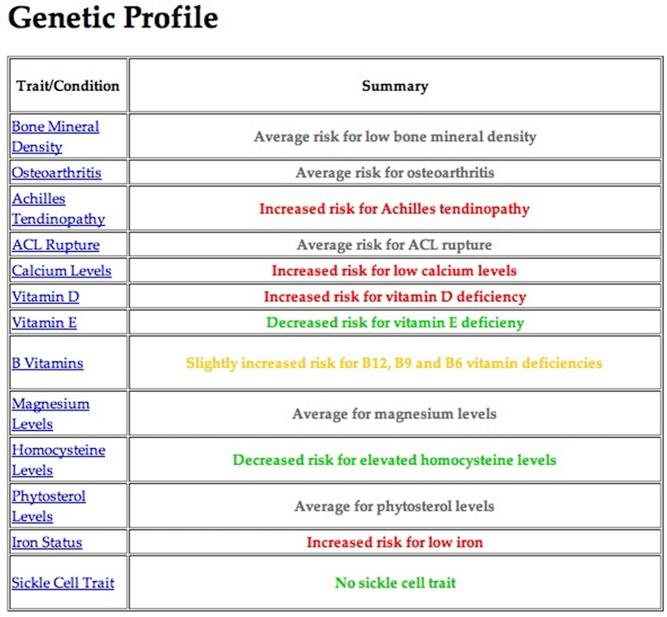
Example Summary of an Athlete's Genetic Profile. Each athlete was given information related to the categories tested. The summary page gives four color-coded risk levels for each trait: decreased risk (green), average (black), slightly increased risk (yellow), or increased risk (red). Further information, including background information, injury mechanism, genetic basis, and prevention strategies was accessible by clicking on the category in the side menu.

We analyzed the raw genotype files for SNPs associated with the six types of injuries identified in the extensive literature review (Table 2). These injury categories were selected for their relevance to athletes (Table 3), and because they are actionable; that is, athletes can modify their training and/or diet to reduce their overall risk. After the raw genotype files were analyzed, athletes went through an hour-long confidential consultation session. If an athlete was found to be at increased risk for a specific injury category, they were given information on preventative measures that could be taken for that specific injury. Fig 2 shows an example of the profiles given to the athletes.

**Table 2:** Summary of Findings from the Genetic Literature Review.

Injury/Trait	Number of SNPs	Effect Size *	Level of Evidence **	Study Type	References
**ACL Rupture**	4	Large	Weak	Candidate Gene	[16–21]
**Achilles Tendon Injuries**	6	Large	Weak	Candidate Gene	[26–31]
**Bone Mineral Density**	67	Large	Strong	GWAS	[41–44]
**Osteoarthritis**	7	Small	Strong	GWAS	[45, 48–49]
**Vitamin/Mineral Deficiencies**	39	-	-	-	-
Iron Biomarkers	8	Small	Moderate	GWAS	[51, 52]
Vitamin E	3	Small	Moderate	GWAS	[53,54]
Vitamin D	6	Medium	Moderate	GWAS	[55, 56]
Calcium	2	Small	Strong	GWAS	[57, 58]
Magnesium	4	Small	Moderate	GWAS	[59]
B Vitamins	7	Medium	Moderate	GWAS	[60, 61]
Homocysteine	8	Small	Moderate	GWAS	[62, 63]
Phytosterols	3	Small	Moderate	GWAS	[64]
**Sickle Cell Trait**	1	Mendelian	Strong	Candidate Gene	[67, 68]

*: Effect size estimates are based on odds ratios reported for published SNPs associated with each category. Odds ratios of 1.0–1.3 are small effects, 1.3–2.0 are medium effects, and greater than 2.0 are large effectsVarsity status is defined as whether or not the athlete participated on an NCAA Div 1, II, or III Varsity level team (swimming, cross-country, track-and-field, crew) for at least 1 year prior to being on the Stanford Triathlon Team.

**: Level of evidence based on criteria for assessment of cumulative evidence of genetic associations from Ioannidis et al 2008 [14].

**Table 3:** Relevance of Selected Injuries and Conditions to Athletes.

Injury/Trait	Relevance to Athletes
**Anterior Cruciate Ligament Rupture**	A severe sports-related injury sustained in multiple landing and cutting/pivoting sports.
**Achilles Tendinopathy**	Common tendinopathies among athletes from repeated overloading of the tendon.
**Low Bone Mineral Density**	A major risk factor for stress fractures and stress reactions among athletes.
**Osteoarthritis**	The onset of osteoarthritis is related to joint injuries, which are common among athletes.
**Vitamin/Mineral Deficiencies**	Minor mineral deficiencies and chronic low vitamin levels can impair athletic performance.
**Sickle Cell Trait**	SCT can cause potentially fatal complications for athletes when training under extreme and stressful conditions.

The first injury category, rupture of the ACL, is one of the most severe injuries sustained in active populations[16]. Candidate gene studies performed on athletes have found SNPs in the *COL1A1*, *COL5A1*, *COL12A1*, and *MMP12* genes that are associated with ACL tears[16,17,18,19,20,21]. Although these studies were performed with small sample sizes, the SNPs associated with ACL rupture have high allelic odds ratios between 2.4 and 50. One Stanford triathlete had a genetic score that indicated protection against ACL tears, while several athletes were found to be at risk. As this injury is rare in endurance athletes, participants did not express concern about their genetic predisposition for ACL tears during their consultations. Dynamic warm-up programs that target proper landing and cutting form, core strength, and hamstring strength have been shown to be effective at reducing ACL injuries[22].

The second injury category, Achilles tendon injury, includes degenerative and painful conditions that affect athletes in a wide range of sports, including up to 18.5% of runners[23,24,25]. Candidate gene studies have found variants within the *MMP3*, *COL5A1*, *COL1A1*, *CASP-8*, and *GDF5* genes to be associated with Achilles tendon pathologies[26,27,28,29,30,31]. Interestingly, the polymorphism rs1800012 is located within a regulatory region of the promoter of *COL1A1*, which encodes type I collagen, the major protein component of tendons and ligaments[32,33]. The rare T allele increases expression of the gene, whereas the G allele lowers expression and is associated with increased risk for Achilles tendon rupture (OR = 11)[18]. Standard practices to prevent Achilles tendon injuries include stretching to increase flexibility, plus eccentric strengthening of the gastrocnemius and soleus muscles[34,35,36]. Three triathletes were identified with a genetic risk for Achilles tendon injuries, two of which had a history of lower leg muscle issues (gastrocnemius and flexor digitorum longus strains). To reduce the risk of an Achilles tendon injury, these athletes began gastrocnemius/soleus eccentric strengthening, calf and Achilles stretches, and shifted to running on softer surfaces.

The third injury category, stress fracture, is a common overuse injury in repetitive sports, and affects up to 20% of female athletes and military recruits[37]. Bone mineral density is a major determinant in a bone’s ability to withstand loading and low BMD is an intrinsic risk factor for stress fractures in athletes[37,38]. It has been shown that genetics determine 50–85% of the variance in BMD[39]. Active, young females reporting a family history of osteoporosis or low BMD were almost twice as likely to develop a stress fracture[40]. Estrada *et al*. performed a large meta-analysis on lumbar spine and femoral neck bone mineral density, including 17 GWA studies involving a total of 32,961 individuals. They identified 64 SNPs associated with bone mineral density at genome-wide significance. An algorithm was developed summing the individual contributions from 63 of these SNPs into one combined genetic score. These scores were divided into five different risk categories based on the population distribution[41]. Individuals in the highest risk category have 1.56 increased odds for osteoporosis, and 1.60 increased odds for fracture. Conversely, individuals in the lowest category were protected against osteoporosis and fracture and had decreased odds of 0.38 and 0.54, respectively[41]. Three additional studies found three SNPs that are also significantly associated with bone mineral density or fracture that were not included in the 2012 Estrada *et al*. paper[42,43,44].

We reported the combined genetic score developed by Estrada *et al*., as well as the individual SNP results, for a total of 66 SNPs. Two participants had genetic scores on the extreme ends of the risk groups established by Estrada *et al*. The triathlete with the worst genetic risk score had a history of three previous stress fractures during his/her career as a collegiate runner. A DEXA scan, which directly measures BMD, indicated osteopenia for this athlete. The triathlete with the most protective score had no history of stress fracture, and only one stress reaction so far as a cross-country runner. Triathletes can easily redistribute training volume to cycling or swimming if they are predisposed to running injuries, such as stress fractures.

The fourth category, osteoarthritis, is a major cause of pain and disability in the elderly and is the most common form of arthritis worldwide[45]. Sports and high-impact activities lead to cartilage lesions, which eventually lead to early-onset osteoarthritis[46,47]. There are seven SNPs associated with osteoarthritis at genome-wide significance[45,48,49]. These SNPs, however, only have allelic odds ratios between 1.1 and 1.2, which indicates a small effect on osteoarthritic risk. Given the strong link between joint injury and subsequent development of osteoarthritis, a focus on preventing joint injuries in young athletes with high risk could prevent or delay the onset of disease. Two Stanford triathletes were heterozygous at all seven loci, which have an additive allele effect [45,48,49]. This information could affect an athlete’s decision regarding: 1) surgical treatment for a joint injury, 2) training plan (intensity or duration) when they are older, and 3) the age at which they retire from competition.

The fifth category includes SNPs associated with vitamin and mineral deficiencies. Exercise results in biochemical adaptations in muscle that place metabolic pathways under stress and increase the need for micronutrients. Routine training increases the loss of micronutrients, thus requiring greater intake of micronutrients for building, maintaining, and repairing lean body mass[50]. Moreover, proper levels of calcium and vitamin D are important for maintaining bone health, while iron, zinc, and the vitamin B complex are important for hematological function.

We reported on 39 SNPs associated with levels of: iron status biomarkers, circulating vitamin E, circulating vitamin D, serum calcium, magnesium, vitamin B12, vitamin B6, vitamin B9, phytosterol, and plasma homocysteine[51,52,53,54,55,56,57,58,59,60,61,62,63,64]. Meta-analyses on iron biomarkers included over 6,600 individuals, and the associated SNPs explain 0.7%-2.2% of the variance in serum iron levels[51,52]. Wang *et al*. performed a GWAS on vitamin D involving 33,996 individuals and found SNPs that explain 1–4% of the variance in serum levels[56]. O'Seaghdha *et al*. performed a GWAS on 20,611 individuals and found SNPs that account for 0.54% of the variance in serum calcium concentrations[57]. Genetic predispositions for vitamin or mineral deficiencies can prompt athletes to consume adequate amounts of micronutrients. Research has shown that many athletes have suboptimal magnesium levels, which can compromise athletic performance[65]. One of the athletes we tested had a genetic risk for magnesium deficiency. As an endurance athlete who needs to replenish micronutrients during activity, this athlete switched to an electrolyte drink that had higher levels of potassium and magnesium.

The last category, sickle cell trait, is a Mendelian condition caused by the inheritance of one normal allele of the hemoglobin gene (hemoglobin A), and one abnormal allele (hemoglobin S, rs334)[66,67,68]. In individuals heterozygous for the hemoglobin β chain mutant allele, red blood cells can sickle when the athlete is subjected to strong stressors, such as high altitude, extreme heat, dehydration, and exhaustion. This sickling can lead to gross hematuria, splenic infarction, and exertional rhabdomyolysis, which can be fatal[69]. No triathlete was positive for sickle cell trait. The NCAA currently recommends that athletes with sickle cell trait engage in slow and gradual conditioning regimens, rest and recover adequately between repetitions, stop activity immediately upon experiencing symptoms, stay optimally hydrated, maintain proper asthma treatment, refrain from exercise during acute illness, use supplemental oxygen as needed, and seek prompt medical care when experiencing unusual distress[70].

Twelve of fourteen athletes were recommended training interventions. The 3-month and 12-month questionnaires showed that eight of those twelve athletes acted upon their genetic results. Two of the remaining four who had not started an intervention indicated that they plan on taking action in the future. Moreover, seven believe that the implemented changes have had, or will have, a positive impact on their athletic careers. Importantly, after one year, four athletes were still actively following their interventions. In the year preceding this pilot study, ten of the fourteen total athletes (71%) sustained an injury (Table 1). However, during the one-year follow up period, only four of the twelve athletes (33%) for whom we had full data sustained an injury (Table 1).

## Discussion

There is a rich compendium of genetic studies that can be used to study sports injury risk in elite athletes. By including genetic studies of health and disease, we have greatly expanded the database of DNA variations that can be applied to sports genetics. We performed a literature search and identified 124 SNPs that have been associated with six different risk categories: ACL rupture, Achilles tendon injury, low bone mineral density, osteoarthritis, vitamin and mineral deficiencies, and sickle cell trait. To our knowledge, 113 of these SNPs have not previously been used in sports genetics[7,8,10,71].

We looked at large genetic studies investigating health traits that could be of interest to athletes. Most published gene association studies investigate disease in the general population rather than athletes. For example, studies on bone mineral density and vitamin/mineral deficiencies have not been previously used in sports genetics. Estrada *et al*. studied low bone mineral density, osteoporosis, and fractures in older women. We propose that these findings may also be relevant for athletes. Training creates a physical stress that, together with genetic predispositions, may lead to an injury that normally only occurs when older. Genetic information linked to vitamin and mineral deficiencies can be useful to help optimize micronutrient intake. Testing for sickle cell trait is already mandatory for all Division I and Division II NCAA athletes. We included the rs334 variant in our report because the DNA test is more accurate and cost-effective than the hemoglobin solubility tests recommended by the NCAA[72].

We began by recruiting elite collegiate triathletes for several reasons. First, triathletes are at increased risk for developing a wide range of injuries due to the three different disciplines of the sport. Second, Stanford University triathletes are club athletes in an individually scored sport, and hence ethical considerations regarding student-athlete scholarships and starting line-ups were not a concern. Athletes were individually motivated to participate (or not) in the genetic testing program, and independently chose whether or not to share their genetic results with third parties. Finally, these athletes are highly dedicated to athletic performance and could benefit from a new competitive edge. Although the genetic associations described here only contribute a small amount to overall risk for injury, they may still be important to elite athletes looking for every edge they can get. Over the 2011 and 2012 ITU World Triathlon Series race seasons, the average time difference between 1^st^ and 10^th^ for the Elite Men was 1.5 minutes, or about 1.4% of the total race time for each event[73]. Optimization of training and diet from individual genetic information could make a difference for some of these athletes.

Nevertheless, the small effect from genetic testing for athletic injury may mean that it is currently premature for use in the general athletic population[74]. Injury etiologies are complex combinations of extrinsic and intrinsic factors. In the future, better understanding of the multifactorial etiologies of sports-related injuries will help guide the use of genetic information in athletic performance and in the clinical management of ‘high-risk’ individuals[75,76].

This pilot program was limited to only fourteen athletes, which is too small to perform a rigorous trial to show the effectiveness of the sports genetics program in reducing injury incidence and to draw meaningful conclusions about the effectiveness of the genetic testing. Our goal was to simply observe the athletes' responses to the new genetic knowledge. Nevertheless, there was positive feedback about the benefits of genetic testing. Fourteen of the nineteen athletes that were contacted decided to participate. Of those that replied to the 3- and 12-month follow up questionnaires, all responded positively to the program and thirteen indicated that they found the genetic results both useful and informative. Moreover, all but one athlete reported sharing their genetic information with their coaches, trainers, physicians and/or teammates, highlighting that genetic information can be used to benefit athletes through multiple channels. Eight out of twelve athletes that were recommended training changes actually modified their training based on their genetic data. Besides prompting athletes to include new modifications in their training or diet, genetic knowledge may also increase compliance with currently prescribed prehabilitation strategies[77]. Genetic testing for sports injury risk could expand to include other types of elite athletes. Long-distance runners and rowers could benefit from this program as these athletes are also at risk for stress fractures, one of the major categories covered by our genetic testing. Knee ligament sprains and Achilles tendon injuries are common to many sports, such as baseball, basketball, soccer, and football. Athletes in these sports could benefit from genetic information regarding ACL rupture and Achilles tendon injury.

## Conclusion

There is a rich source of new genetic knowledge that can be applied to athletic injury risk. Many health risks, such as low bone mineral density in the elderly, may also be pertinent to athletes. Athletes push themselves to the limit, which increases their chance for injury, and small effects in physiology can have meaningful consequences in competition. Through our literature search, we identified 113 SNPs that have previously not been discussed in relation to sports injuries and performance. A new sports genetic database was constructed, and used to help customize training and injury prevention for fourteen triathletes. In the future, additional categories of injury can be included in the sports genetics database, and personalized genetic consultation can be expanded to other types of athletes.

## Supporting Information

S1 File Three- and Twelve-month Follow-up Survey Questions. Text copy of survey questions distributed to athletes three and twelve months after initial results consultation.
(PDF)
