# Correction: Applying Personal Genetic Data to Injury Risk Assessment in Athletes

**DOI:** 10.1371/journal.pone.0171397

**Published:** 2017-01-27

**Authors:** Gabrielle T. Goodlin, Andrew K. Roos, Thomas R. Roos, Claire Hawkins, Sydney Beache, Stephen Baur, Stuart K. Kim

There is an error in the Ethics Statement under the Materials and Methods section. The correct Ethics Statement is: The pilot program protocol was approved by the Research Compliance Office at Stanford University (IRB Protocol 24576), with the exception of the post-consultation surveys, which were not submitted to or reviewed by the IRB. We have confirmed with our IRB that the survey portion of the research, included as Supporting Information S1 File, was not human subjects research. Written informed consent was obtained from all athletes to participate in the study, as well as verbal consent to use their deidentified information for study purposes.
