# Supplementary material for: Applying Personal Genetic Data to Injury Risk Assessment in Athletes
Source: PLoS One. 2015 Apr 28;10(4):e0122676. doi: 10.1371/journal.pone.0122676 (PMC4412532; doi:10.1371/journal.pone.0122676)
Supplement: S1 File — Text copy of survey questions distributed to athletes three and twelve months after initial results consultation. (PDF) [file pone.0122676.s001.pdf]

Stanford Triathlon Athlete Survey 12mon

Please complete the survey below.

Thank you!

Athlete Injury History

Before participating in this pilot study, did you have a medical diagnosis or a previous history of any of the following conditions/injuries? Please report only injuries for which you missed 1 or more days of training.

- ☐ Achilles Tendon Injury
- ☐ ACL Injury
- ☐ Ligament or Tendon Injury (other)
- ☐ Soft Tissue Injury (other)
- ☐ Osteoarthritis
- ☐ Low Bone Mineral Density
- ☐ Stress Fracture
- ☐ Fracture (other)
- ☐ Muscle Injury
- ☐ Overuse Injury or Tendonitis
- ☐ Iron Deficiency
- ☐ Vitamin or Mineral Deficiency (other)
- ☐ Sickle Cell Trait
- ☐ None of the above
- ☐ Decline to Respond

(Select all that apply.)

How many workouts did you do on average per week over the last year (01/01/2013 to 01/01/2014)? Please report unique training sessions that were separated by at least 1hr from the previous workout. Ie. A brick workout where you combine 2 disciplines back-to-back counts as 1 workout.

____
(Enter the # of workouts rounded up to a whole number.)

How many hours did you train on average per week over the last year (01/01/2013 to 01/01/2014)? Please report the average over your training season, excluding the off-season.

____
(Enter the # of hours rounded up to a whole number.)

Were you injured during the 2012-13 season (Sept 2012 to Aug 2013)? An injury is defined as missing 1 or more days of training.

- ☐ Yes
- ☐ No

(Select if you had any injuries that prevented you from training or competing for at least 1 day.)

Please indicate any/all diagnosed injuries that you had during the 2012-13 season. Please report only injuries for which you missed 1 or more days of training.

- ☐ Achilles Tendon Injury
- ☐ ACL Injury
- ☐ Ligament or Tendon Injury (other)
- ☐ Soft Tissue Injury (other)
- ☐ Stress Fracture
- ☐ Fracture (other)
- ☐ Muscle Injury
- ☐ Overuse Injury or Tendonitis
- ☐ Other Injury

(Select all that apply.)

Were you injured during the 2013-14 season (Sept 2013 to Jan 2014)? An injury is defined as missing 1 or more days of training.

- ☐ Yes
- ☐ No

(Select if you had any injuries that prevented you from training or competing for at least 1 day.)

Please indicate any/all diagnosed injuries that you had during the 2013-14 season. Please report only injuries for which you missed 1 or more days of training.

- ☐ Achilles Tendon Injury
- ☐ ACL Injury
- ☐ Ligament or Tendon Injury (other)
- ☐ Soft Tissue Injury (other)
- ☐ Stress Fracture
- ☐ Fracture (other)
- ☐ Muscle Injury
- ☐ Overuse Injury or Tendonitis
- ☐ Other Injury

(Select all that apply.)

Intervention Information Usage

Please indicate any/all of the prevention programs that were recommended to you.

- ☐ Achilles Tendon Injury
- ☐ ACL Injury
- ☐ Bone Mineral Density & Stress Fracture
- ☐ Osteoarthritis
- ☐ Vitamin/Mineral Deficiency
- ☐ Sickle Cell Trait
- ☐ None

(Select all that apply.)

Please indicate any/all of the prevention programs that you adopted into your training plan after participating in this program.

- ☐ Achilles Tendon Injury
- ☐ ACL Injury
- ☐ Bone Mineral Density & Stress Fracture
- ☐ Osteoarthritis
- ☐ Vitamin/Mineral Deficiency
- ☐ Sickle Cell Trait
- ☐ None

(Select all that apply.)

How many days elapsed between your consultation meeting and any actions you took regarding your results?

____
(Enter the # of days rounded up to a whole number.)

For how long did you actively follow an intervention after you consultation meeting?

- ☐ I did not take any action and do not plan to in the future
- ☐ I have not taken any action yet, but plan to in the future
- ☐ 1-7 days
- ☐ 1-2 weeks
- ☐ 2-4 weeks
- ☐ 1-2 months
- ☐ 2-6 months
- ☐ I am still following my program, but am unsure if I will continue in the future
- ☐ I am still following my program, and will continue in the future

(Select the most appropriate choice.)

Genetic Information Usage

Have you kept your genetic results confidential?

- ☐ I have kept my results confidential, and do not plan on sharing them in the future.
- ☐ I have kept my results confidential, but plan on sharing them in the future.
- ☐ I originally kept my results confidential, but since the last survey have shared them.
- ☐ I shared my results before the last survey, but no longer will share my results in the future.
- ☐ I shared my results after the consultation, and plan on continuing to share my results in the future.

(Select the most appropriate choice.)

Did you share your genetic risk information with any of the following?

- ☐ Coach(es)
- ☐ Athletic Trainer(s)
- ☐ Physician/Doctor(s)
- ☐ Physical Therapist(s)
- ☐ Teammate(s)
- ☐ None of the above

(Please select all that apply.)

Please select your level of agreement with the following statements about how your genetic results have affected your interactions with other people.

	Strongly Disagree	Disagree	Neutral	Agree	Strongly Agree	Not Applicable
My genetic results changed the way I interact with my coach(es).	☐	☐	☐	☐	☐	☐
My genetic results changed the way I interact with my athletic trainer(s).	☐	☐	☐	☐	☐	☐
My genetic results changed the way I interact with my physician/doctor(s).	☐	☐	☐	☐	☐	☐
My genetic results changed the way I interact with my physical therapist(s).	☐	☐	☐	☐	☐	☐
My genetic results changed the way I interact with my teammate(s).	☐	☐	☐	☐	☐	☐

Please select your level of agreement with the following statements about your genetic results.

	Strongly Disagree	Disagree	Neutral	Agree	Strongly Agree
I believe that the information about injury prevention is interesting.	☐	☐	☐	☐	☐
I believe that the information about injury prevention is useful.	☐	☐	☐	☐	☐
My genetic results changed the way I think about my training.	☐	☐	☐	☐	☐
My genetic results changed the way I actually train.	☐	☐	☐	☐	☐
I believe the intervention program(s) has had an impact already on my training and/or competing.	☐	☐	☐	☐	☐
I believe the intervention program(s) will have an impact in the future on my training and/or competing.	☐	☐	☐	☐	☐

Please select how your genetic results have affected you.

	Had no effect	Had a small effect	Had a moderate effect	Had a large effect
What effect did your genetic results have on your view of yourself as an athlete?	☐	☐	☐	☐
What effect did your genetic results have on your confidence while training and/or competing?	☐	☐	☐	☐
What effect did your genetic results have on your motivation to train?	☐	☐	☐	☐
What effect did your genetic results have on your motivation to race?	☐	☐	☐	☐
What effect did your genetic results have on the likelihood that you will continue participating in competitive sports in the future?	☐	☐	☐	☐
